# Beyond visual integration: sensitivity of the temporal-parietal junction for objects, places, and faces

**DOI:** 10.1186/s12993-024-00233-2

**Published:** 2024-04-18

**Authors:** Johannes Rennig, Christina Langenberger, Hans-Otto Karnath

**Affiliations:** 1 Division of Neuropsychology, Center of Neurology, Hertie-Institute for Clinical Brain Research, University of Tübingen, D-72076 Tübingen, Germany; 2 Department of Psychology, University of South Carolina, Columbia, USA

**Keywords:** TPJ, Visual perception, Object, Face, Place

## Abstract

One important role of the TPJ is the contribution to perception of the global gist in hierarchically organized stimuli where individual elements create a global visual percept. However, the link between clinical findings in simultanagnosia and neuroimaging in healthy subjects is missing for real-world global stimuli, like visual scenes. It is well-known that hierarchical, global stimuli activate TPJ regions and that simultanagnosia patients show deficits during the recognition of hierarchical stimuli and real-world visual scenes. However, the role of the TPJ in real-world scene processing is entirely unexplored. In the present study, we first localized TPJ regions significantly responding to the global gist of hierarchical stimuli and then investigated the responses to visual scenes, as well as single objects and faces as control stimuli. All three stimulus classes evoked significantly positive univariate responses in the previously localized TPJ regions. In a multivariate analysis, we were able to demonstrate that voxel patterns of the TPJ were classified significantly above chance level for all three stimulus classes. These results demonstrate a significant involvement of the TPJ in processing of complex visual stimuli that is not restricted to visual scenes and that the TPJ is sensitive to different classes of visual stimuli with a specific signature of neuronal activations.

## Highlights

Left and right hemispheric TPJ regions show comparable BOLD univariate responses to different object classes (objects, faces, places).

Demonstration that the TPJ has unique activation patterns for the different object classes.

Specifically positive activations for TPJ regions are significantly involved in global perception compared to TPJ regions not responding to global shapes.

Above chance level decoding of objects, faces, and places from TPJ regions are involved in global perception.

## Introduction

The temporo-parietal junction (TPJ) is involved in various cognitive functions, like understanding other people’s intentions and behavior (Theory of Mind; Saxe and Kanwisher [11, 44, 75, 76], visual search and orienting of attention [14, 28, 38, 39] and visual stimulus detection [5]. Studies investigating patients with damage to bilateral temporo-parietal cortices exhibiting simultanagnosia and functional imaging studies suggested a TPJ involvement in perception of hierarchical global stimuli [2, 25, 32, 48], and objects in demanding viewing conditions [13, 58, 69, 71].

Several studies that associated temporo-parietal brain regions with global perception of hierarchical stimuli used Navon-like stimuli [56] where a global percept is constructed from local elements [29, 33, 67, 68, 85]. However, a Navon-like, hierarchical stimulus was always intended as a representation of real-world visual scenes where individual elements create a global visual precept e.g., humans, trees, walkways, and grass create the global scene impression of a park. It is also known that patients suffering from simultanagnosia show significant deficits in perception of global, hierarchical stimuli and grasping the gist of visual scenes like the Broken Window Picture [3, 35, 66, 72].

A recent fMRI study [58] showed the connection between clinical observations in simultanagnosia and functionality of the healthy human brain. It was demonstrated that the TPJ responds particularly to objects presented in demanding viewing conditions which is in line with patient studies reporting particular deficits for demanding object presentations in simultanagnosia [13, 66]. However, the link between clinical findings in simultanagnosia and neuroimaging in healthy subjects is missing for real-world global stimuli, like visual scenes. While it is well-known that Navon-like, hierarchical stimuli used as artificial representations of real-world global scenes activate TPJ regions [29, 33, 67, 68, 85] and that simultanagnosia patients show deficits during the recognition of hierarchical stimuli and visual scenes the role the TPJ in real-world scene processing is unclear.

In the current study, we aimed at investigating the role of the TPJ in processing of real-word scenes. We first conducted a fMRI TPJ localizer experiment [33] to identify voxels in the TPJ responding to global, hierarchical stimuli. In an independent fMRI experiment, we showed real-world scenes as well as objects and faces as control stimuli. We hypothesized that TPJ voxels that respond to artificial representations of global scenes should show stronger responses to real-world visual scenes compared to objects and faces. While previous research already showed an involvement of the TPJ in processing of visual scenes [55], human faces [46], and object use [83] the present study aims at finding differences in univariate activations or unique voxel patterns between the three stimulus classes (see below).

Beyond univariate responses, multivariate voxel patterns in selected regions of interest provide a unique and sensitive insight into the functionality of a particular brain region. We hypothesized that TPJ areas (responding to global shapes) of the healthy human brain should show unique voxel patterns for real-world scenes, and possibly real-world complex objects and faces. The existence of unique voxel patterns for real-world scenes (and other stimulus classes) would show evidence for specific mechanisms supporting perception of different stimulus classes with different perceptual requirements. Eventually, this result would help to understand the mechanisms causing simultanagnosia where lesions in posterior temporo-parietal brain regions gradually impair the perception of hierarchical stimuli, real-world visual scenes or even coherent, complex objects. Since the perceptual impairments observed in simultanagnosia are usually no all-or-nothing phenomenon (e.g., modulated by severity of the task, Huberle & Karnath [32, 66], with partial damage to crucial brain regions specific activation pattern might be able to compensate deficits under certain conditions. In our multivariate analysis approach, we trained a machine learning classifier to assess the specificity of voxel response patterns in the TPJ to real-world scenes, objects, and faces.

## Methods

18 healthy individuals participated in the experiments (6 left-handed and 7 female; mean age = 26 years, SD = 3). All had normal or corrected-to-normal vision and gave written informed consent prior to scanning. Participants reported no history of neurological or psychiatric disorders. The experiment was approved by the ethics committee of the medical faculty of the University in Tübingen and conducted in accordance with the declaration of Helsinki.

The sample size was chosen based on prior experience with fMRI studies involving the TPJ and visual processing providing the necessary statistical power [7, 33, 58, 67, 68].

MRI scans were acquired using a 3T Siemens Magnetom Trio scanner (Siemens AG, Erlangen, Germany), using a 64-channel head coil. Stimuli were presented using Matlab (The Mathworks, Inc., Natick, MA, USA) and the Psychophysics Toolbox [9, 62] and shown on a MR compatible screen placed behind the scanner bore which could be viewed by the participants via a mirror mounted on the head coil. Behavioral responses were collected using a fiber-optic button response pad (Current Designs, Haverford, PA, USA).

**Fig. 1:**
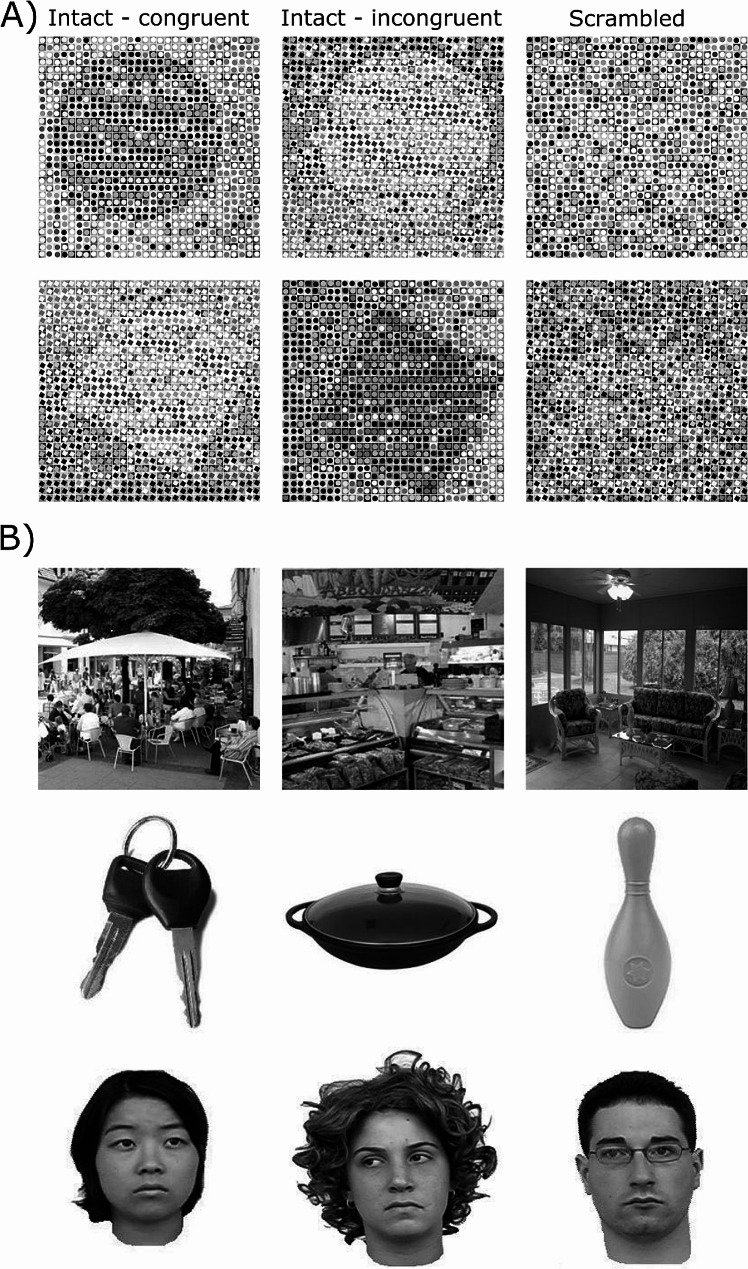
Stimulus material. **(A)** In the localizer fMRI experiment, we showed the global shapes of either a circle or a square constructed from local images of squares or circles. All possible combinations of global and local elements were presented. The images were scrambled at two levels (20% and 80%) so that the global form could either be recognized (intact global shapes) or not (scrambled global shapes). **(B)** In the main fMRI experiment, we presented visual stimuli from three different categories: places, objects, and faces

In the *fMRI localizer experiment*, we presented Navon-like global shape stimuli [56] that were applied in previous neuroimaging studies [7, 33, 58, 67, 68]. The stimuli showed the global shape of either a circle or a square constructed from local images of squares or circles and were presented in all possible combinations of global and local elements (congruent and incongruent). Each stimulus consisted of 900 small elements organized in 30 columns and 30 rows. In order to minimize learning effects, all global objects were presented at one of four different positions within an individual stimulus (left top, right top, left bottom, right bottom) and luminance and contrast were varied between the objects and their background (e.g., dark objects presented in a light background and vice versa). We created 192 different stimuli (4 combinations of objects at the global and local level, 48 stimuli per combination differing in luminance and position of the global objects). The stimulus images were scrambled at two levels (20% and 80%) so that the global form could either be recognized (‘intact global perception’) or not (‘scrambled global perception’; Fig. 1A). The stimuli were scrambled by exchanging the small images of objects at the local level with each other. The percentage number indicated hereby the percentage of relocated local elements in relation to their total number. Stimuli were presented in two runs (duration of each block: about 7 min), each consisting of 168 experimental trials (42 intact circles, 42 intact squares, 42 scrambled circles, 42 scrambled squares). The 168 stimuli were selected pseudo-randomly from the set of 192 stimuli available keeping a balanced number of intact and scrambled global stimuli. The global forms were presented at 5.4° visual angles (size of the background square of each stimulus). Participants were instructed to respond via button press whether the stimulus was a circle or a square.

In the *main fMRI experiment*, we used visual stimuli of three different classes: places, objects and faces (Fig. 1B). We choose classic place stimuli as the representation of visual scenes. The original images were taken from multiple sources [8, 41, 42, 59, 61, 70, 74]. Since the three stimulus classes have systematic differences, e.g. faces are mostly round, objects can have various shapes, we controlled for low-level stimulus features. In the first step, all stimulus classes were converted to grayscale using the *rgb2gray* function in Matlab. In a next step, object and face stimuli were normalized for size by extending the stimuli on their longer axis on the image background and adjusting the other axis accordingly, i.e., if an object/face was higher than wide we stretched it to fully cover the y-axis of the background image and adjusted stimulus size accordingly on the x-axis. Luminance was adjusted across all stimuli by calculating the average pixel value across all stimuli and adjusting the pixel values of each stimulus to the average pixel value. For object and face stimuli only the non-white proportions of the stimulus were considered to calculate the luminance per individual stimulus and across stimuli. To control for differences in spatial frequencies between stimulus classes we calculated spatial frequencies across all stimuli and excluded the images with the 10% lowest and highest values. We did not filter any spatial frequencies from the images since this would induce another systematic bias, e.g., through blurring, and might cause an unnatural appearance of a stimulus. All stimuli were presented at a size of 5° visual angles (size of the background square of each stimulus). The stimuli were selected from the databases in a way that preferably few similar objects were included. Four runs with a duration of 8 min were conducted. Per run, 180 experimental trials were presented (60 per class) in a pseudorandomized order. Twelve stimuli (four from each stimulus class) were repeated, and stimulus repetitions had to be indicated via button press in a one-back task. In total, 673 different stimuli were presented throughout the experiment.

We investigated the image features contrast, luminance, and spatial frequency between the three stimulus classes for all stimuli used in the present study. We calculated a linear model per image feature with the predictor *stimulus type* (objects, faces, places) and the respective feature values of the individual stimuli as dependent variable found significant differences between all three stimulus types for contrast (*F* = 12.03, *p* = 7.9 × 10^− 6^), luminance (*F* = 55.07, *p* < 2.0 × 10^− 16^) and spatial frequency (*F* = 866.56, *p* < 2.0 × 10^− 16^). However, each stimulus type showed particularly different patterns across image features. Place stimuli showed the highest value for spatial frequencies (*p* < 2.0 × 10^− 16^ compared against faces and objects). In contrast, object stimuli had the highest luminance (*p* < 2.0 × 10^− 16^ compared against faces and *p* = 0.0953 compared against places) and face stimuli the highest contrast (*p* = 9.0 × 10^− 6^ compared against objects and *p* = 6.7 × 10^− 7^ compared against places). In conclusion, each of the three stimulus types showed image characteristics varying unsystematically from the other stimulus types which makes it unlikely that these image features systematically influenced BOLD responses in non-visual areas of the brain, the TPJ.

Both fMRI experiments were event-related designs and stimuli were presented for 300 ms with an inter-stimulus interval of 1700 ms. During the inter-stimulus interval, a central fixation crosshair was presented. The events were ordered in an optimal rapid event-related design specified by *optseq2* [15]; https://surfer.nmr.mgh.harvard.edu/optseq) adding an additional fixation baseline time (80 s in the *fMRI localizer experiment* and 120 s in the *main fMRI experiment*), distributed between the trials.

### MRI data acquisition

Functional images were acquired using multiband echo-planar-imaging (EPI) sequences. All remaining participants were scanned with parameters for multiband EPI sequences from the HCP [53]: TR = 1000 ms, TE = 37 ms, flip angle = 52°, FOV = 187 × 187 mm^2^, 72 slices, voxel size = 2 × 2 × 2 mm^3^. Single band reference images (TR = 1000 ms, TE = 37 ms, flip angle = 52°, FOV = 1872 × 1872 mm^2^, 72 slices, voxel size = 2 × 2 × 2 mm^3^) were collected before each functional run. Per participant, two T1-weighted anatomical scans (TR = 2280 s, 176 slices, voxel size = 1.0 × 1.0 × 1.0 mm^3^; FOV = 256 × 256 mm², TE = 3.03 ms; flip angle = 8°) were collected at the end of the experimental session.

### fMRI data analysis

Data pre-processing and model estimation were performed using *SPM12* (http://www.fil.ion.ucl.ac.uk/spm). Functional images were realigned to each participant’s first image, aligned to the AC-PC axis and slice-time corrected. The original single-band image was then co-registered to the pre-processed functional images and the anatomical image was co-registered to the single-band image. The resolution of the single-band image was up-sampled before the anatomical image was aligned to it. Functional images were smoothed with a 4 mm FWHM Gaussian kernel. Time series of hemodynamic activation were modeled based on the canonical hemodynamic response function (HRF) as implemented in SPM12. Low-frequency noise was eliminated with a high-pass filter of 128 s. Correction for temporal autocorrelation was performed using an autoregressive AR(1) process. Movement parameters (roll, pitch, yaw; linear movement into x-, y-, z-directions) estimated in the realignment were included as regressors of no interest. To avoid bias associated with spatial normalization, analyses were conducted in native space. For the *fMRI localizer experiment*, we used two experimental regressors: *intact global shapes* and *scrambled global shapes*. For the *main fMRI experiment*, the experimental regressors consisted of the three stimulus classes: *places, objects*, and *faces*. Hit trials from the one-back task and trials with accidental/erroneous button presses were not modeled explicitly. To give MNI-coordinates of our functional ROIs we normalized the anatomical and functional data and re-created the functional ROIs in MNI space identical to the method described below in native space.

### Region of interest (ROI) analysis

Anatomical ROIs were created applying Freesurfer’s cortical reconstruction routine [16, 22] and the Destrieux atlas [19] for each subject. To create an individual anatomical TPJ ROI for each participant, we combined the posterior third of the superior temporal gyrus (Freesurfer Label 11,174 and 12,174), the sulcus intermedius primus (Freesurfer Label 11,165, 12,165), the angular gyrus (Freesurfer Labels 11,125, 12,125) and the posterior half of the supramarginal gyrus (Freesurfer Label 11,126, 12,126). Since the functional anatomy of global perception is widely under debate within posterior temporo-parietal brain areas with possibly high inter-individual variability we decided to create individual global TPJ ROIs within liberal anatomical boundaries [7, 58]. Our individual anatomical-functional ROIs might therefore represent an adequate anatomical correlate of global perception in individual posterior temporo-parietal brain areas.

**Fig. 2:**
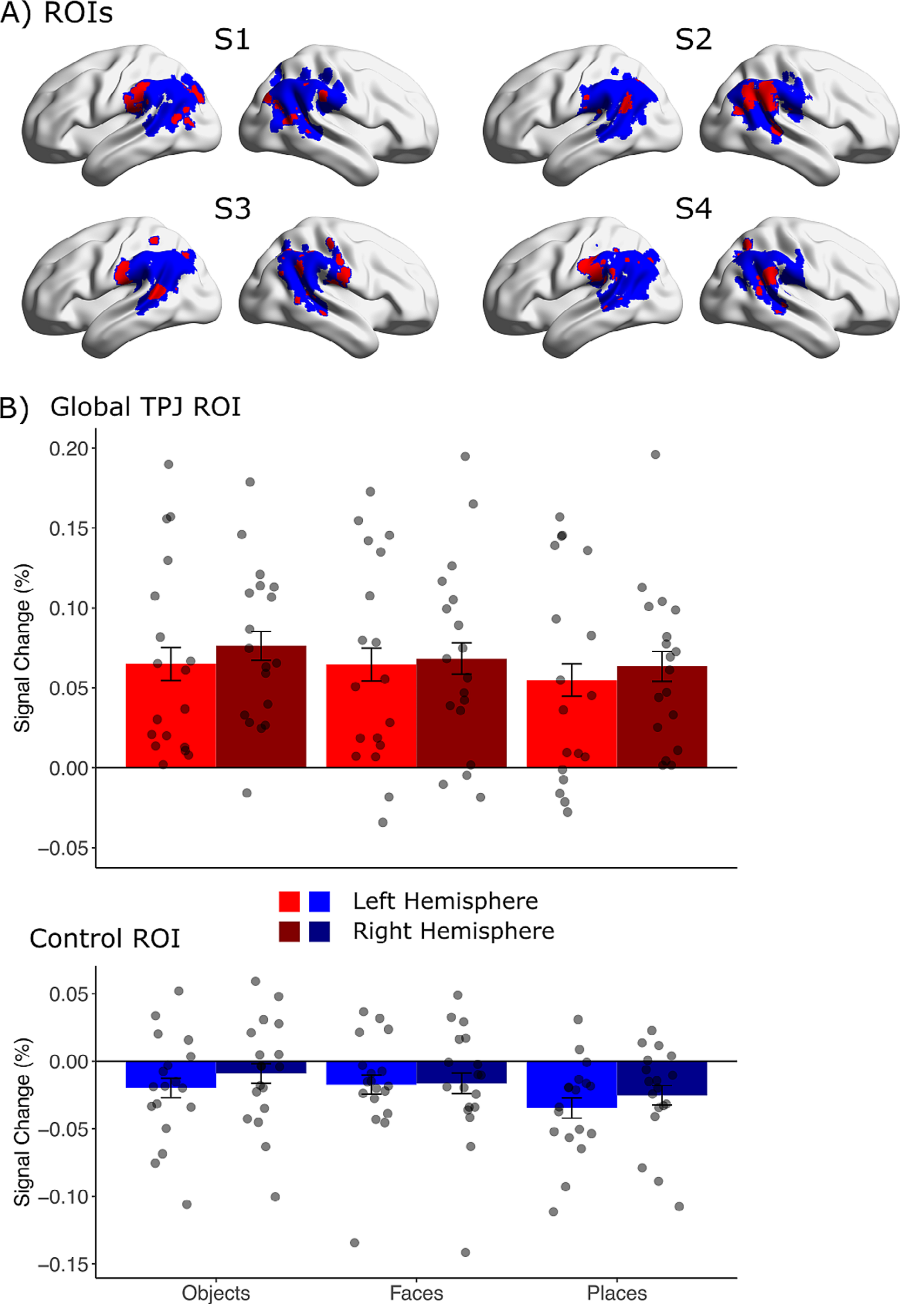
Univariate ROI Analysis **(A)** Individual anatomical TPJ ROIs were created applying Freesurfer’s cortical reconstruction routine [16, 22] and the Destrieux atlas [19]. From these ROIs, we then identified voxels that showed higher signals for intact global shapes compared to baseline for each subject (red voxels: global TPJ ROI, blue voxels: control ROI). Percent signal change values and beta-coefficients extracted from these individual ROIs were then used for univariate and multivariate statistical analyses. Example ROIs from four representative subjects are presented in in standard MNI space on a surface version of the ch2 brain using the BrainNet viewer [84]. **(B)** Percent signal change values for places, objects, and faces from left and right hemispheric global shape TPJ ROIs with corresponding error bars (standard error of the mean). The dots represent the individual data points per participant

In a next step, we identified individual voxels that showed higher signals for intact global shapes compared to fixation baseline as functional ROIs involved in global shape perception. The voxel-level threshold was set to *p* < 0.05 (uncorr.) without cluster threshold. Each participant’s individual global shape TPJ ROI was created as an intersection between the functional intact global shapes vs. baseline contrast and the anatomical TPJ ROI. We were able to identify functional global shape TPJ ROIs in all our 18 participants in the left and right hemisphere. Example structural and functional TPJ ROIs are presented in Fig. 2A. The average size of the individual TPJ global shape ROIs was 1216.22 mm^3^ (SD = 902.07 mm^3^) in the left hemisphere and 1027.78 mm^3^ (SD = 515.95 mm^3^) in the right hemisphere. The mean center of mass was located at the MNI coordinates x = −48.33 (SD = 6.48); y = −41.67 (SD = 9.76); z = 32.78 (SD = 9.26) for left hemispheric global shape TPJ ROIs and x = 47.56 (SD = 4.68); y = −44.67 (SD = 8.03); z = 31.11 (SD = 8.71) for right hemispheric global shape TPJ ROIs. Since the ROIs were created based on individual anatomy and individual BOLD responses to the functional localizer task they differed substantially in size and form also allowing partially connected or distinct subclusters (see Fig. 2A).

As control ROI we selected all voxels of the individual, anatomical TPJ ROIs not responding to global shapes (Fig. 2A). The average size of these individual control ROIs was 11104.20 mm^3^ (SD = 1493.74 mm^3^) in the left hemisphere and 11562.70 mm^3^ (SD = 1479.70 mm^3^) in the right hemisphere. The mean center of mass was located at the MNI coordinates x = −49.44 (SD = 2.15); y = −51.44 (SD = 2.73); z = 29.44 (SD = 2.55) for left hemispheric and x = 51.00 (SD = 1.85); y = −46.33 (SD = 3.77); z = 30.00 (SD = 3.36) for right hemispheric ROIs.

We used *MarsBar* (http://marsbar.sourceforge.net) to extract the mean percent signal change from the individual global shape TPJ ROIs for all three experimental conditions of the *main fMRI experiment* (per run and participant). For statistical data analysis we applied linear mixed effect models using *R*’s *lme4* and *lmerTest* packages. Model estimation was done using Restricted Maximum Likelihood (REML) estimation. Statistical significance was assessed using the *Anova* function provided by the *car* package.

### Multivoxel pattern analysis (MVPA)

A univariate analysis can only demonstrate differences in signal strengths of experimental conditions in a ROI, e.g., stronger signals for places vs. objects in TPJ regions. In contrast, a MVPA allows to distinguish between multivariate patterns evoked by distinct between experimental conditions [26, 27]. First, we created feature vectors from fMRI data by applying the approach suggested by Mumford et al. [54] for each stimulus class from the *main fMRI experiment* and the *fMRI localizer experiment* and every participant we calculated beta regression coefficient images for each experimental trial separately by running a general linear model including a regressor for the respective trial as well as another regressor for all other trials. For this analysis, we used unsmoothed images and did not apply any high-pass filtering in the statistical model. Resulting beta values of voxels from individual global shape TPJ ROIs (and control ROIs) were then used as features for training and testing support vector machines (SVM) using the *R* package *e1071*.

We aimed at demonstrating that TPJ areas responding to intact global shapes show specific voxel pattern responses for places in contrast to objects and faces. Per participant and hemisphere, we selected beta values for every experimental trial from the *main fMRI experiment* (objects, faces, places) from every voxel of the previously defined global shape TPJ ROI. Each experimental trial was treated as an observation and each voxel as a feature for the machine learning model. We split the data across all experimental runs randomly into a training set (80% of trials = 576 data points) and a test set (20% of trials = 144 data points) and trained an SVM with a *linear* basis kernel with the training set. Using the training data, we conducted grid search using 10-fold cross validation to optimize the regularization parameter C = [0.01, 0.1, 1, 10, 100] and gamma = [0.1, 0.5, 1, 2]. Using the built-in *tune()* function of the *e1071* package for cross-validation we sampled trials randomly from the training data avoiding a systematic bias that can be induced by a leave-one-run-out cross validation. Using the SVM model, we predicted from the voxel patterns of the test set (per participant and hemisphere) if an individual trial was an object, face, or place. We calculated classification accuracy and predictive value per condition. The predictive value per condition was calculated as follows: number of correct classifications (condition A) / [number of correct classifications (condition A) + incorrect classification (condition A)]. The overall classification accuracy is calculated as the proportion of correctly classified trials relative to all classified trials across both object viewing conditions: [correct classifications (condition A) + correct classifications (condition B) + correct classifications (condition C)] / [correct and incorrect classifications (condition A) + correct and incorrect classifications (condition B) + correct and incorrect classifications (condition C)]. We interpret a potential difference in classification accuracies between the two conditions as noisier data in one condition compared to the other condition.

To explore possible differences between a linear and a non-linear representation of stimulus activation patterns we repeated the MVPA with a *radial* basis kernel (with otherwise identical methods and parameters as with the *linear* SVM kernel). This analysis was motivated by the conceptual reasoning that a linear decodability of information indicates an abstract encoding of this information in the respective brain area beyond low-level object features, like shape or size [45, 52]. A linear representation (with higher classification accuracies for a *linear* kernel) would indicate that a brain region encodes high level stimulus concepts beyond simple low-level features.

### Whole brain analysis

We used the spatially normalized functional data to calculate the following whole brain contrasts across all participants: intact vs. scrambled global perception, objects vs. faces and places, faces vs. objects and places as well as places vs. objects and faces. We used the *AAL3* toolbox for *SPM12* [73] to extract the overlap of the clusters from the respective contrasts with the *AAL3* regions.

## Results

### Behavioral data

To ensure attention to the visual stimuli during the *main fMRI experiment* participants were instructed to indicate stimulus repetitions via button press in a one-back task. We calculated a linear mixed-effect model with percent correct values as dependent variable, *stimulus* (objects, faces, places) as fixed effects and *participant* as a random effect. Participants detected repetitions objects (mean: 90%, SD: 14), faces (mean 94%, SD: 6) and places (mean 88%, SD: 11) reliably. We observed no significant main effect for *stimulus* (*χ²* = 3.29, *p* = 0.193).

During the *localizer fMRI experiment*, participants were instructed to indicate whether a stimulus showed a circle or a square. For intact global stimuli, responses were mostly correct (mean: 97%, SD: 16), for scrambled global stimuli, responses were around chance level (mean: 46%, SD: 50).

### Univariate ROI analysis

We observed positive BOLD signals for all three experimental conditions in the global TPJ ROI (Fig. 2B): objects (mean percent signal change left hemisphere: 0.06%; right hemisphere: 0.08%), faces (left hemisphere: 0.06%; right hemisphere: 0.07%) and places (left hemisphere: 0.05%; right hemisphere: 0.06%). In contrast, we observed only deactivations in the remainder of the anatomically defined TPJ (Fig. 2B). We found negative BOLD signals for objects (left hemisphere: -0.02%; right hemisphere: -0.01%), faces (left hemisphere: -0.02%; right hemisphere: -0.02%) and places (left hemisphere: -0.03%; right hemisphere: -0.03%).

To statistically quantify the differences between stimulus classes and ROIs we used percent signal change values as a dependent variable in a linear mixed-effects model with fixed effects for *stimulus* (objects, faces, places), *ROI* (global TPJ ROI, control ROI) and *hemisphere* (left vs. right) and *participant* and *run* as a random effect. There was a significant main effect of *stimulus* (*χ²* = 7.20, *p* = 0.027), *ROI* (*χ²* = 399.72, *p* = 2.0 × 10^− 16^) and no effect for *hemisphere* (*χ²* = 3.05, *p* = 0.081) and no significant interaction (*p* > 0.678). See Table 1 for the full model output. This model was calculated to demonstrate the significant differences in univariate activation between the global TPJ ROI and the control ROI.

**Table 1:** Parameter estimates and results of the univariate ROI analysis (comparison between ROIs)

	Estimate	Std. Error	df	t	p	
**Fixed effects**						
(Intercept)	0.06	0.01	47.78	5.64	8.9 × 10^− 7^	
Faces	0.00	0.01	842.90	−0.04	0.97	
Places	−0.01	0.01	842.90	−0.97	0.33	
Right Hemisphere	0.01	0.01	842.90	1.08	0.28	
Control ROI	−0.08	0.01	842.90	−8.07	2.6 × 10^− 15^	
Faces × Right Hemisphere	−0.01	0.01	842.90	−0.52	0.60	
Places × Right Hemisphere	0.00	0.01	842.90	−0.18	0.86	
Faces × Control ROI	0.00	0.01	842.90	0.20	0.84	
Places × Control ROI	0.00	0.01	842.90	−0.30	0.76	
Right Hemisphere × Control ROI	0.00	0.01	842.90	−0.04	0.97	
Faces × Right Hemisphere × Control ROI	0.00	0.02	842.90	−0.10	0.92	
Places × Right Hemisphere × Control ROI	0.00	0.02	842.90	0.06	0.96	
*Random effects*	*Variance*					
Participant	0.001					
Run	0.000					
*Main effects*	*χ²*	*p*				
Stimulus	7.19	0.027				
ROI	399.77	2.0 × 10^− 16^				
Hemisphere	3.05	0.081				
*Interactions*						
Stimulus × ROI	0.32	0.852				
Stimulus × Hemisphere	0.78	0.678				
ROI × Hemisphere	0.01	0.922				
Stimulus × ROI × Hemisphere	0.02	0.987				

We used mean percent signal change values for places, objects and faces from individual global shape TPJ ROIs and control ROIs as dependent variable. *Stimulus, ROI*, and *hemisphere* were set as fixed effects and *participant* and *run* were set as random effects in a linear mixed-effects model. The upper rows show the parameter estimates for each factor in the model as well as the variance of the random effects. The lower rows show the results of the chi-square test of the model, with one degree of freedom for each factor

To assess statistical differences between stimulus classes and hemispheres in the global TPJ ROI we used percent signal change values as a dependent variable in a linear mixed-effects model with fixed effects for *stimulus* (objects, faces, places) and *hemisphere* (left vs. right) and *participant* and *run* as a random effect. There was no significant main effect of *stimulus* (*χ²* = 2.19, *p* = 0.335) and *hemisphere* (*χ²* = 1.51, *p* = 0.220) and no significant interaction (*χ²* = 0.25, *p* = 0.882). The full model output is presented in Table 2.

**Table 2:** Parameter estimates and statistical results of the univariate ROI analysis (global TPJ ROI)

	Estimate	Std. Error	df	t	p
**Fixed effects**					
(Intercept)	0.06	0.01	32.97	4.58	6.3 × 10^− 5^
Faces	0.00	0.01	410.90	−0.03	0.97
Places	−0.01	0.01	410.90	−0.92	0.36
Right Hemisphere	0.01	0.01	410.90	1.02	0.31
Faces × Right Hemisphere	−0.01	0.02	410.90	−0.49	0.62
Places × Right Hemisphere	0.00	0.02	410.90	−0.17	0.87
*Random effects*	*Variance*				
Participant	0.003				
Run	0.000				
*Main effects*	*χ²*	*p*			
Stimulus	2.20	0.335			
Hemisphere	1.50	0.220			
*Interactions*					
Stimulus × Hemisphere	0.25	0.882			

We used mean percent signal change values for places, objects and faces from individual global shape TPJ ROIs as dependent variable. *Stimulus* and *hemisphere* were set as fixed effects and *participant* and *run* were set as random effects in a linear mixed-effects model. The upper rows show the parameter estimates for each factor in the model as well as the variance of the random effects. The lower rows show the results of the chi-square test of the model, with one degree of freedom for each factor

We calculated the same model for the Control ROI and observed a significant main effect of *stimulus* (*χ²* = 7.53, *p* = 0.023) but not for *hemisphere* (*χ²* = 2.03, *p* = 0.154) and no significant interaction (*χ²* = 0.77, *p* = 0.679). See the full model output in Table 3. Pairwise comparisons showed a significant difference between places and objects (*χ²* = 6.39, *p* = 0.011), places and faces (*χ²* = 4.45, *p* = 0.035) but not between objects and faces (*χ²* = 0.15, *p* = 0.694). This analysis shows that the significant main effect for *stimulus* in the first analysis (including the factor *ROI*) is driven by greater deactivation for *places* in the control ROI and therefore not relevant for the analysis of the global TPJ ROI.

**Table 3:** Parameter estimates and statistical results of the multivariate ROI analysis (global TPJ ROI)

	Estimate	Std. Error	df	t	p
**Fixed effects**					
(Intercept)	36.11	3.06	101.56	11.80	< 2 × 10^− 16^
Faces	6.25	4.08	90.00	1.53	0.13
Places	5.79	4.08	90.00	1.42	0.16
Right Hemisphere	7.41	4.08	90.00	1.82	0.07
Faces × Right Hemisphere	−7.06	5.77	90.00	−1.22	0.22
Places × Right Hemisphere	−12.38	5.77	90.00	−2.15	0.03
*Random effects*	*Variance*				
Participant	19.00				
*Main effects*	*χ²*	*p*			
Stimulus	0.61	0.736			
Hemisphere	0.01	0.922			
*Interactions*					
Stimulus × Hemisphere	4.64	0.098			

We used individual predictive values for places, objects and faces as dependent variable. *Stimulus* and *hemisphere* were set as fixed effects and *participant* was set as random effect in a linear mixed-effects model. The upper rows show the parameter estimates for each factor in the model as well as the variance of the random effects. The lower rows show the results of the chi-square test of the model, with one degree of freedom for each factor

### MVPA

**Fig. 3:**
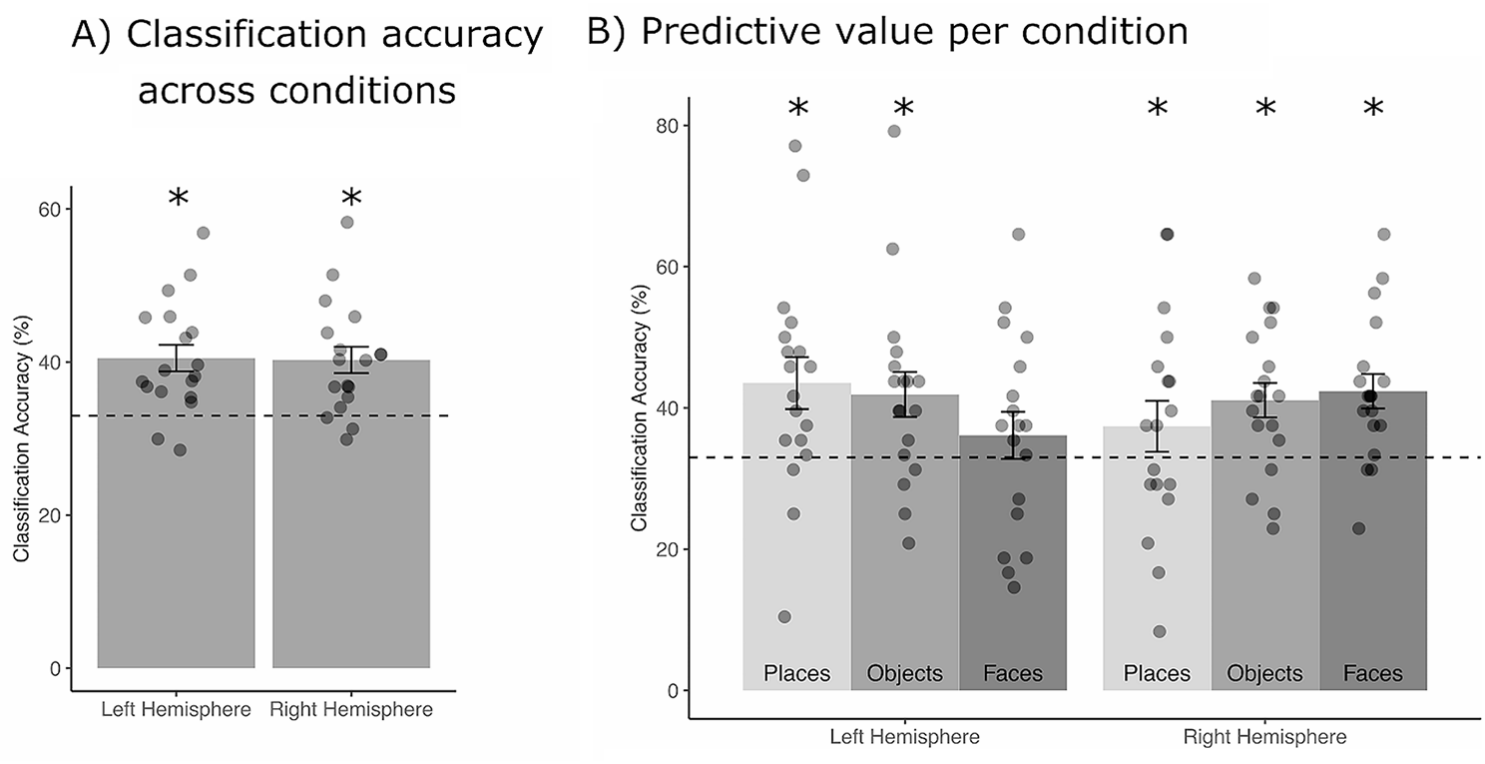
Multivoxel Pattern Analysis (MVPA). **(A)** Average classification accuracy in the global TPJ ROI across all three stimulus conditions (places, objects, faces) in the left and right hemisphere. (**B)** Average predictive values (calculated as the proportion of correctly classified trials relative to all tested trials for all three stimulus classes) for places, objects, and faces in left and right hemispheric global shape TPJ ROIs. In both panels, the error bars are the standard error of the mean. The dashed line indicates the 33% chance level. The asterisk indicates significant results against 33% chance level. The dots represent the individual data points per participant

In the left and right hemispheric global TPJ ROI, the overall classification accuracy was significantly above chance level (Fig. 3A; *left hemisphere*: mean: 40.5%; *t*-test against 33% chance level: *t*_*(17)*_ = 4.34, *p* = 4.4 × 10^− 5^; *right hemisphere*: 40.2%; *t*_*(17)*_ = 6.15, *p* = 4.8 × 10^− 7^).

In the left hemispheric global TPJ ROI (Fig. 3B), we observed predictive values significantly above chance for objects (41.9%; *t*_*(17)*_ = 2.80, *p* = 0.018) and places (43.5%; *t*_*(17)*_ = 2.86, *p* = 0.018), but not for faces (36.1%; *t*_*(17)*_ = 0.94, *p* = 0.363). The predictive values in the right hemispheric global TPJ ROI (Fig. 3B) were significantly above chance for objects (41.1%; *t*_*(17)*_ = 4.31, *p* = 3.8 × 10^− 4^), faces (42.4%; *t*_*(17)*_ = 2.97, *p* = 0.007) and places (37.4%; *t*_*(17)*_ = 2.88, *p* = 0.007).

All *t*-tests of classification accuracies against chance are corrected for multiple comparisons using the *False Discovery Rate (FDR)* per MVPA analysis.

To assess statistical differences of SVM classification between stimulus classes and hemispheres we used predictive values as a dependent variable in a linear mixed-effects model with fixed effects for *stimulus* (objects, faces, places) and *hemisphere* (left vs. right) and *participant* as a random effect. There was no significant main effect of *stimulus* (*χ²* = 0.361 *p* = 0.736) and *hemisphere* (*χ²* = 0.01, *p* = 0.922) and no significant interaction (*χ²* = 4.64, *p* = 0.098). See Table 3 for the full model output.

To statistically investigate the differences in classification accuracies between SVM models applying a *linear* vs. *radial* kernel (in the global TPJ ROI) we calculated a linear mixed-effects model with predictive values (each stimulus class) as a dependent variable fixed effects for *kernel* (linear, radial), *stimulus* (objects, faces, places) and *hemisphere* (left vs. right) and *participant* as a random effect. We observed higher classification accuracies for the *linear* kernel throughout all stimulus classes and both hemispheres (see Table 4) and a significant main effect for *kernel* (*χ²* = 13.23, *p* = 2.8 × 10^− 4^) indicating a significant difference. There was no other significant main effect (*p* > 0.106) and no significant interaction (*p* > 0.125). See Table 5 for the full model output.

**Table 4:** Predictive values per kernel, hemisphere and stimulus class (global TPJ ROI)

	Linear kernel		Radial kernel	
	left	right	left	right
Objects	42.0 (13.5)	41.1 (10.3)	38.2 (11.0)	36.1 (10.7)
Faces	36.1 (14.1)	42.4 (10.4)	30.9 (10.0)	31.8 (10.5)
Places	43.5 (15.6)	37.4 (15.3)	36.6 (8.5)	34.4 (12.4)

Predictive values per kernel, hemisphere and stimulus class in mean percentage values with SDs in brackets across all participants from the global TPJ ROI. A linear mixed-effects model showed a significant main effect for *kernel* (*χ²* = 13.23, *p* = 2.8 × 10^− 4^) indicating a significant difference between predictive values across all stimulus types and hemispheres

**Table 5:** Parameter estimates and results of the multivariate ROI analysis (comparison of linear and radial kernels)

	Estimate	Std. Error	df	t	p
**Fixed effects**					
(Intercept)	36.11	2.76	214.67	13.08	< 2 × 10^− 16^
Kernel Radial	−5.21	3.86	198.00	−1.35	0.18
Objects	5.79	3.86	198.00	1.50	0.14
Places	7.41	3.86	198.00	1.92	0.06
Hemisphere	6.25	3.86	198.00	1.62	0.11
Kernel Radial × Objects	1.51	5.46	198.00	0.28	0.78
Kernel Radial × Places	−1.74	5.46	198.00	−0.32	0.75
Kernel Radial × Hemisphere	−5.32	5.46	198.00	−0.98	0.33
Objects × Hemisphere	−7.06	5.46	198.00	−1.29	0.20
Places × Hemisphere	−12.38	5.46	198.00	−2.27	0.02
Kernel Radial × Objects × Hemisphere	4.05	7.72	198.00	0.53	0.60
Kernel Radial × Places × Hemisphere	9.26	7.72	198.00	1.20	0.23
*Random effects*	*Variance*				
Participant	3.26				
*Main effects*	*χ²*	*p*			
Kernel	13.23	2.8 × 10^− 4^			
Stimulus	4.50	0.106			
Hemisphere	0.18	0.668			
*Interactions*					
Kernel × Stimulus	0.95	0.622			
Kernel × Hemisphere	0.08	0.778			
Stimulus × Hemisphere	4.16	0.125			
Kernel × Stimulus × Hemisphere	1.45	0.485			

We used individual predictive values for objects, faces and places as dependent variable. *Kernel*, *stimulus* and *hemisphere* were set as fixed effects and *participant* was set as random effect in a linear mixed-effects model. The upper rows show the parameter estimates for each factor in the model as well as the variance of the random effects. The lower rows show the results of the chi-square test of the model, with one degree of freedom for each factor

We repeated the MVPA for the control ROI using a *linear* kernel and compared the classification accuracies between the two ROIs (global TPJ ROI vs. control ROI). In the left and right hemispheric control ROIs, the overall classification accuracy was above chance level but not significant (*left hemisphere*: mean: 35.8%; *t*-test against 33% chance level: *t*_*(17)*_ = 2.07, *p* = 0.054; *right hemisphere*: 34.9%; *t*_*(17)*_ = 1.37, *p* = 0.207). In the left hemispheric control ROI, we observed predictive values above chance (but not significant) for objects (36.8%; *t*_*(17)*_ = 1.39, *p* = 0.182), places (33.5%; *t*_*(17)*_ = 0.14, *p* = 0.891) and faces (37.1%; *t*_*(17)*_ = 1.32, *p* = 0.205). The predictive values in the right hemispheric control ROI were also not significantly above chance for objects (34.0%; *t*_*(17)*_ = 0.28, *p* = 0.780), faces (33.6%; *t*_*(17)*_ = 1.99, *p* = 0.845) and places (37.2%; *t*_*(17)*_ = 1.37, *p* = 0.190). Next, we compared predictive values between the global TPJ ROI and the control ROI. We used predictive values as a dependent variable in a linear mixed-effects model with fixed effects for *ROI* (global TPJ ROI vs. control ROI), *stimulus* (objects, faces, places) and *hemisphere* (left vs. right) and *participant* as a random effect. We observed a significant main effect of *ROI* (*χ²* = 8.63, *p* = 0.003) but no other significant main effects or interactions (*p* > 0.061). The full model output is presented in Table 6.

**Table 6:** Parameter estimates and results of the multivariate ROI analysis (comparison of global TPJ ROI and control ROI)

	Estimate	Std. Error	df	t	p
**Fixed effects**					
(Intercept)	37.17	3.07	203.83	12.09	< 2 × 10^− 1^
ROI	−1.06	4.18	198.00	−0.25	0.80
Objects	−0.35	4.18	198.00	−0.08	0.93
Places	−3.70	4.18	198.00	−0.89	0.38
Hemisphere	−3.59	4.18	198.00	−0.86	0.39
global TPJ ROI × Objects	6.13	5.92	198.00	1.04	0.30
global TPJ ROI × Places	11.11	5.92	198.00	1.88	0.06
global TPJ ROI × Hemisphere	9.84	5.92	198.00	1.66	0.10
Objects × Hemisphere	0.81	5.92	198.00	0.14	0.89
Places × Hemisphere	7.29	5.92	198.00	1.23	0.22
Kernel Radial × Objects × Hemisphere	−7.87	8.37	198.00	−0.94	0.35
Kernel Radial × Places × Hemisphere	−19.68	8.37	198.00	−2.35	0.02
*Random effects*	*Variance*				
Participant	3.26				
*Main effects*	*χ²*	*p*			
ROI	8.63	0.003			
Stimulus	0.31	0.858			
Hemisphere	0.11	0.743			
*Interactions*					
ROI × Stimulus	0.28	0.870			
ROI × Hemisphere	0.04	0.848			
Stimulus × Hemisphere	0.63	0.729			
ROI × Stimulus × Hemisphere	5.60	0.061			

We used individual predictive values for objects, faces and places as dependent variable. *ROI*, *stimulus* and *hemisphere* were set as fixed effects and *participant* was set as random effect in a linear mixed-effects model. The upper rows show the parameter estimates for each factor in the model as well as the variance of the random effects. The lower rows show the results of the chi-square test of the model, with one degree of freedom for each factor

### Whole brain analysis

**Fig. 4:**
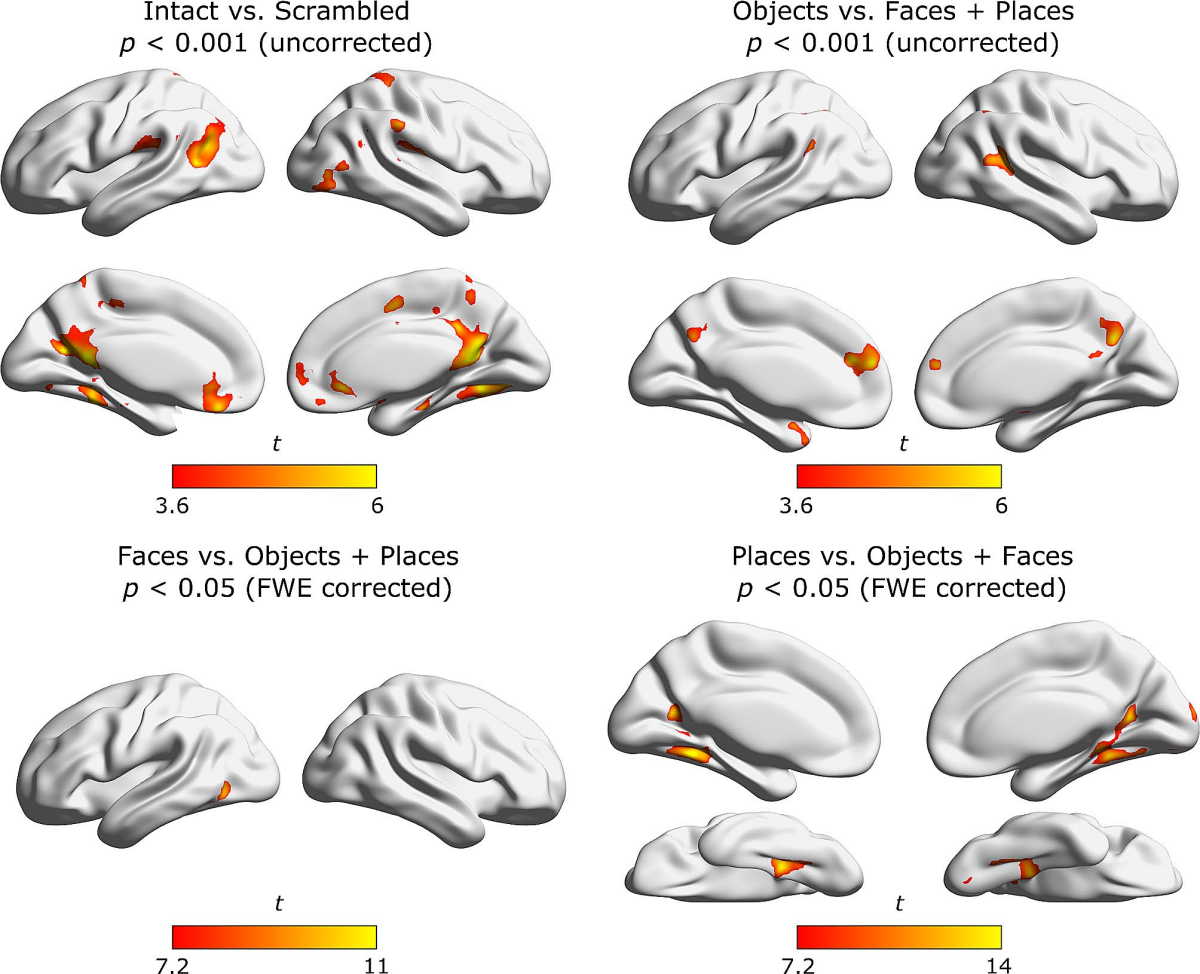
Whole brain results. Whole brain results presented in in standard MNI space on a surface version of the ch2 brain using the BrainNet viewer [84]. The color bar indicates the *t* value of a certain voxel for the respective contrast. For the contrasts Intact vs. Scrambled global perception and Objects vs. Faces and Places no voxel survived a FWE correction for multiple comparisons

For the contrasts intact vs. scrambled global perception and objects vs. faces and places we only report results uncorrected for multiple comparisons (*p* < 0.001). For the contrasts faces vs. objects and places as well as places vs. objects and faces a considerable number of voxels and clusters survived a correction for multiple comparisons (*p* < 0.05, FWE). For all contrasts we set a cluster threshold of 100. Results from the whole brain analysis are shown in Table 7; Fig. 4.

**Table 7:** Location of cluster peaks from all whole brain contrasts in MNI space and % overlap with left and right hemispheric AAL regions

x	y	z	Hemisphere	AAL Label	% overlap
**Intact vs. Scrambled,** *** p*** ** < 0.001 (uncorrected)**					
0	−10	46	L	Cingulate_Post	66.31
−6	36	−18	L	Frontal_Med_Orb	29.90
−50	−58	10	L	Angular	27.20
0	−10	46	L	Cingulate_Mid	23.96
0	−10	46	L	Precuneus	16.07
−26	−40	−6	L	Lingual	14.61
−50	−20	18	L	SupraMarginal	12.58
0	−10	46	L	Cuneus	12.29
−50	−20	18	L	Rolandic_Oper	11.03
−50	−58	10	L	Temporal_Mid	10.50
−26	−40	−6	L	ParaHippocampal	10.22
0	−10	46	R	Cingulate_Post	62.09
0	−10	46	R	Cingulate_Mid	23.33
22	−70	−2	R	Fusiform	22.52
50	−16	12	R	Rolandic_Oper	21.26
−6	36	−18	R	Frontal_Med_Orb	17.52
0	−10	46	R	Precuneus	16.78
50	−16	12	R	Heschl	12.45
22	−70	−2	R	Lingual	11.52
22	−70	−2	R	ParaHippocampal	11.40
50	−16	12	R	SupraMarginal	10.89
22	−70	−2	R	Occipital_Inf	10.62
**Objects vs. Places + Faces**, ***p*** **< 0.001 (uncorrected)**					
−32	−52	40	L	Parietal_Inf	11.20
−34	2	−20	L	Temporal_Pole_Sup	10.66
−2	52	18	L	Frontal_Sup_Medial	10.53
6	−58	32	L	Precuneus	10.20
6	−58	32	R	Cingulate_Post	14.03
44	−50	40	R	Parietal_Inf	10.04
**Faces vs. Places + Objects**, ***p*** **< 0.05 (FWE corrected)**					
−48	−64	−6	L	Occipital_Mid	20.98
−48	−64	−6	L	Occipital_Inf	15.30
−48	−64	−6	L	Fusiform	15.20
46	−58	−4	R	Occipital_Mid	14.54
**Places vs. Faces + Objects**, ***p*** **< 0.05 (FWE corrected)**					
−26	−48	−8	L	ParaHippocampal	16.87
−26	−48	−8	L	Lingual	10.84
24	−42	−10	R	ParaHippocampal	18.29
24	−42	−10	R	Lingual	14.35
24	−42	−10	R	Fusiform	12.91

The percent overlap value indicates the amount of overlap of the respective AAL with the whole brain results. Only regions with > 10% overlap are shown

## Discussion

In the present study, we were able to show that bilateral TPJ regions contribute to perception of several independent object stimulus classes (objects, faces, places) with unique activation patterns coding each stimulus class specifically. We demonstrated that TPJ regions involved in perception of global, hierarchical structures are also active during perception of coherent objects of several object classes. This suggests that both, hierarchical organized stimuli and coherent objects, are being processed in similar bilateral TPJ regions. While univariate responses were significantly positive, but not different between the three object classes (objects, faces, places), we identified unique activation patterns for each object class in our multivariate analysis. This suggests that the TPJ may contribute with a specific strategy to the processing of each object class.

Our results are in line with previous work showing a significant contribution of the TPJ in the perception of coherent objects [37, 43, 58, 77, 80, 81]. A recent study by Nestmann and colleagues [58] demonstrated that TPJ regions, predominantly in the left hemisphere, responded significantly positive to object stimuli. Here, TPJ regions of interest were functionally localized using hierarchical global shapes [7, 58, 67, 68] and object stimuli of different viewing conditions were presented. In contrast, anatomically defined TPJ regions that did not respond to global shapes showed a significantly negative response. With the present study, we were able to replicate and extend the results of Nestmann et al. [58]. We demonstrated that the TPJ is not only involved in the perception of objects stimuli but also involved in processing of faces and places with a unique signature of neuronal activations for each object class.

Our results are also in good agreement with studies in simultanagnosia that demonstrated significant impairments with perception deficits for hierarchical stimuli as well as objects, scenes and places [13, 17, 57, 66]. While simultanagnosia patients do not only suffer from impairments perceiving hierarchical stimuli like Navon-letters [56] they also show significant problems grasping the gist of visual scenes like the Broken Window Picture [3, 35, 66, 72]. The present study confirms these clinical observations by demonstrating that TPJ regions that encode global structures also contribute generally (significant univariate responses) and specifically (unique voxel patterns) to the perception of object classes where simultanagnosia patients show deficits with, e.g. coherent objects, faces and visual scenes [13, 57, 66]. In conclusion, it is valid to assume the existence of a distributed network of TPJ voxels that contribute to global perception and perception of coherent structures that - when lesioned - cause symptoms of simultanagnosia.

Our results are in line with several neuroimaging studies that have demonstrated dorsal contributions to object perception [18, 23, 24, 40]. Numerous studies showed significant contributions of the posterior temporal sulcus which was included into our anatomical ROI definition in face [63–65] and social scene perception [36, 82]. However, the present study used non-social scenes (without displaying human interaction) adding inanimate scenes to the categories of stimuli being processed through the TPJ.

A recent study [1] demonstrated an interesting contribution of dorsal brain areas during the perception of objects and their local elements. In a localizer experiment, the authors identified bilateral anterior and posterior regions in the intraparietal sulcus (IPS) that processed object-centered relations of local object parts. In a next step, it was demonstrated that the object category (e.g., boats, cars) from an independent experiment was successfully decoded from the right posterior IPS. This result shows the crucial and specific contributions of dorsal brain areas to highly specific processes of object perception. However, the study by Ayzenberg and Behrmann [1] also suggests the possibility for a more fine-grained and potentially more informative approach to investigate scene perception in posterior temporo-parietal brain areas, e.g., modulating visual scenes composed of different numbers of local objects to directly test the involvement of the TPJ in hierarchical scene perception.

Our results are also supported by several studies applying TMS to the TPJ providing evidence from a method allowing causal conclusions beyond functional neuroimaging that cannot support casual claims. It was shown that inhibitory TMS to the TPJ significantly reduced the ability for mental rotation of face stimuli indicating a significant representation of visually presented faces in the TPJ region [86]. It was also demonstrated that a semantic advantage in object processing was significantly disturbed through TMS inhibition over the TPJ suggesting a higher order representation of objects in this this brain area [60]. A study using movies as visual scene stimuli (designed to test mechanisms of the Theory of Mind) showed that predictions about future events of that presented scene were significantly influenced by TMS to the TPJ [4]. All three studies support our findings of significant and specific contributions of the TPJ to face, object and scene processing and suggest a particular high-level mechanism of visual perception for all three stimulus types facilitated through TPJ regions.

Our analysis investigating differences between *linear* and *radial* kernels of the SVM models demonstrated significantly better classification accuracies for the SVM model using the *linear* kernel. This result, with better decoding of the linear classifier, indicates a high-level, abstract representation of each object class in TPJ regions. This linear representation shows that TPJ regions in fact encode high level stimulus concepts beyond simple low-level object features [45, 52].

We hypothesized that place stimuli might elicit the strongest responses in the TPJ compared to objects and faces. The hypothesis was based on clinical observations in simultanagnosia patients that struggle the most with scene/place stimuli compared to single objects [72] and the fact that every real-world visual scene where individual elements create a global visual entity represents a hierarchical global stimulus where single elements create a superior percept. However, there were no significant differences between places, objects, and faces, but the MVPA detected unique activation signatures across all TPJ voxels responding to global shapes for all three stimulus classes. The behavioral deficits in global scene perception known to be typical for simultanagnosia could therefore arise from problems interpreting an incomplete activation pattern due to lesions to the TPJ that is specific to the patterns of place stimuli. Another reason for the similar univariate activations across all three stimulus classes could be the general functionality of the TPJ as brain area providing the necessary resources of visual attention to all kinds of object-like visual stimuli [5, 31]. A higher sensitivity for hierarchical Navon-like shapes [29, 33, 34, 67, 68] and objects in demanding viewing conditions [13, 57, 58, 66] might also be explained by a higher attentional demand in the TPJ for more complex object-like visual content.

Another possible explanation for the absence of significant differences in the univariate analysis could be the complexity of otherwise coherent objects also requiring significant contributions from posterior temporo-parietal brain areas [13, 57, 58, 66]. This explanation is in line with the Recognition-by-Components Theory [6] postulating that objects are visually processed assembling various local components to a superior, coherent percept. A comparable explanation can be applied for face stimuli that per se can be seen as hierarchically organized entities where local elements, like mouth, eyes, and nose, create a superior percept. Several studies suggested a holistic, Gestalt-like processing of faces in healthy human participants [30, 78, 79]; for a review see Maurer et al [50]. , while a study with patients suffering from simultanagnosia also showed significant deficits in face recognition [49, 51]. Taken together, the absence of a significant difference between neuronal signals for places, objects and faces can be explained by the possibly similar visual processing mechanisms for all three stimulus types.

The results of the present study fit very well with the Recognition-by-Components Theory [6] postulating that real-world objects are assembled from various local components to a superior, coherent percept. While it was previously shown that posterior temporo-parietal brain areas in close vicinity of the TPJ [29, 33, 67, 68] are involved in processing of hierarchically organized visual stimuli [56] a mechanism of general feature integration for real-world objects is a possible explanation for the results found in our univariate and multivariate analyses.

Another popular theory of visual perception, the theory of visual attention (TVA) [10], and especially one of its extensions, the contour detector (CODE) theory of visual attention [47] might help explaining the present results. The CODE theory claims that visual attention clusters nearby items into perceptual groups which applies to individual objects and their subcomponents as well as relations of objects in space. This mechanism that is very similar to visual integration known from processing of hierarchically organized Navon-like stimuli [56] and can therefore help to explain the present results. Since the TPJ was reported to be involved in visual search and orienting of attention [14, 28, 38, 39] the CODE theory fits well with our results showing a connection between the TPJ and rather perceptual mechanisms processing different kinds of real-world object types.

A possible limitation of the current study is the focus on posterior temporo-parietal brain regions located in close vicinity to the TPJ [7, 33, 58, 67, 68]. Neuroimaging studies [20, 21] and studies with simultanagnosia patients [12, 57] showed a significant involvement of more posterior brain regions in more occipital or ventral areas in mechanisms of global perception. Therefore, significant differences between stimulus conditions in the univariate analysis could possibly be discovered focussing on more posterior/ventral areas as ROIs.

In conclusion, we here demonstrated that the TPJ responds to several kinds of object stimuli expanding expectations from clinical observations in simultanagnosia. A multivariate analysis showed that TPJ subregions that respond to global shapes have a unique activation pattern for places, objects, and faces. These results allow new important insights into the functionality of the TPJ in visual perception and hint towards a general role of the TPJ as a brain area significantly supporting visual perception.

## Data Availability

The fMRI data and analysis scripts (univariate and multivariate analysis) are available under the following URL: https://osf.io/v8w7f. The structural and functional scans are not publicly available due to the data protection agreement of the University of Tübingen, as approved by the ethics committee of the medical faculty of the University of Tübingen and signed by the participants. Scans are available on request to the corresponding author following a formal data sharing agreement after obtaining informed consent of each participant.
